# Association of lifetime lactation and characteristics of menopause: a longitudinal cohort study

**DOI:** 10.1186/s12889-024-20508-7

**Published:** 2024-11-11

**Authors:** Natalie V. Scime, Beili Huang, Meredith Merilee Brockway, Hilary K. Brown, Erin A. Brennand

**Affiliations:** 1 Department of Health and Society, University of Toronto Scarborough, Toronto, ON Canada; 2 Department of Obstetrics and Gynecology, University of Calgary, Calgary, AB, Canada; 3 Faculty of Nursing, University of Calgary, Calgary, AB Canada; 4 Dalla Lana School of Public Health, University of Toronto, Toronto, ON Canada

**Keywords:** Breastfeeding, Menopause, Oophorectomy, Hysterectomy, Childbirth, Longitudinal study, Alberta’s Tomorrow Project

## Abstract

**Background:**

Lactation has many established benefits for women’s long-term health; however, its influence on menopause is less clear. This study investigated the association between lifetime duration of lactation and the timing and type of menopause in midlife women.

**Methods:**

We analyzed survey data on 19,783 parous women aged 40 to 65 years at enrollment in the Alberta’s Tomorrow Project (2000–2022), a prospective community-based cohort study in Alberta, Canada. Duration of lifetime lactation across all births was categorized as: <1 month (reference group; 19.8% of women), 1–3 months (12.1%), 4–6 months (11.7%), 7–12 months (18.8%), and ≥ 13 months (37.7%). Women were classified as premenopause, natural menopause (age at 1 year after the final menstrual period), surgical menopause (age at bilateral oophorectomy), or indeterminate menopause (age at premenopausal hysterectomy with ovarian preservation). Flexible parametric survival analysis and multinomial logistic regression were used to analyze menopause timing and type, respectively, according to lactation status and controlling for birth year, education, parity, hormonal contraceptive use, and smoking.

**Results:**

In a dose-response manner, longer lactation was associated with reduced risk of natural menopause before age 50 (for ≥ 13 months of lactation, adjusted hazard ratio at age 45: 0.68, 95% CI 0.59–0.78), surgical menopause before age 55 (age 45: 0.56, 0.50–0.63), and indeterminate menopause before age 50 (age 45: 0.75, 0.69–0.82). Longer lactation was associated with lower odds of surgical (adjusted odds ratio 0.54, 95% CI 0.45–0.66) and indeterminate menopause (0.63, 0.55–0.73), compared to natural menopause.

**Conclusions:**

Optimizing the timing of natural menopause and reducing risks of early surgical and indeterminate menopause may be novel maternal benefits of breastfeeding.

**Supplementary Information:**

The online version contains supplementary material available at 10.1186/s12889-024-20508-7.

## Background

Breastfeeding has positive life course effects on maternal health. Cumulative duration of lactation is associated with lowered risk of cardiometabolic disease [1, 2] and reproductive cancers [3, 4], generally in an inverse dose-response manner. Women’s health benefits of breastfeeding are thought to be facilitated by the distinct endocrine profile of lactation and its broader systemic impacts. High prolactin levels inhibit the release of gonadotropins and sex steroid hormones, which modulate several reproductive (e.g., ovulation) and metabolic (e.g., glucose homeostasis) functions [5, 6]. Robust evidence on the lasting impacts of lactation on maternal health can therefore bolster public health promotion of breastfeeding and guide clinical risk stratification of women as they age.

The impact of lifetime lactation duration on menopause is less clear. Menopause demarcates the end of female reproductive function and typically occurs with a final menstrual period in the absence of obvious medical cause between 45 and 58 years, termed natural menopause [7]. Menopause can also be induced medically through removal of the ovaries (oophorectomy) or uterus (hysterectomy).

Given that the female ovarian reserve is fixed at birth, the prevailing “follicle sparing” hypothesis posits that breastfeeding-related changes in endogenous hormones which suppress ovulation and ovarian activity leading to lactational amenorrhea may slow the rate of follicle decline and delay the onset of natural menopause [8–10]. However, studies investigating lactation history among a set of potential factors related to timing of natural menopause have yielded mixed findings [11–19]. Recent prospective studies on lactation as a primary exposure suggest its influence on natural menopause may change as women age; lifetime duration of lactation appears to be associated with reduced risk of early natural menopause before age 45 [20], but not with differences in menopausal timing more broadly (i.e., 45 to 60 years) [21]. These contrasting findings signify a need for evidence on the possible time-varying impact of lactation history across the full continuum of natural menopause and aging. There is also a lack of evidence on medical types of menopause, despite biological pathways between lactation and estrogen-sensitive gynecologic conditions such as endometriosis [22], adenomyosis [23], and uterine fibroids [24] that are frequently treated with oophorectomy or hysterectomy. We sought to generate this evidence by investigating the association between lifetime duration of lactation and the timing and type of menopause in midlife women using a flexible survival analysis approach.

## Methods

### Study sample

We conducted a secondary analysis of the Alberta’s Tomorrow Project (ATP), a province-wide prospective cohort study investigating chronic disease etiology [25]. In total, 52,810 English-speaking Albertans (*n* = 34,950 females) aged 35–69 years with no cancer history were recruited in two phases: two-stage telephone random digit dialing mapped to regional health authorities (2000–2009); and volunteer sampling through targeted communication and advocacy strategies (2009–2015). Comprehensive health and demographic data were collected through standardized questionnaires approximately every 3–5 years with response rates of 70–80%; questionnaires can be found at https://myatpresearch.ca/survey-questions/. For this analysis, we used self-report data from all questionnaires completed by August 2022, representing a median of 2 study contacts (interquartile range 2 to 4 contacts) and 6 years of follow-up (interquartile range 2.7 to 10.7 years) over midlife and beyond per participant. The ATP Study was approved by the Health Research Ethics Board of Alberta at Alberta Innovates (HREBA.CC-17-0461 and HREBA.CC-17-0494) and all participants provided written informed consent. This secondary analysis of ATP data was approved by the Conjoint Health Research Ethics Board at the University of Calgary (REB22-0742) in accordance with the Canadian Tri-Council Policy Statement on Ethical Conduct for Research Involving Humans.

**Fig. 1:**
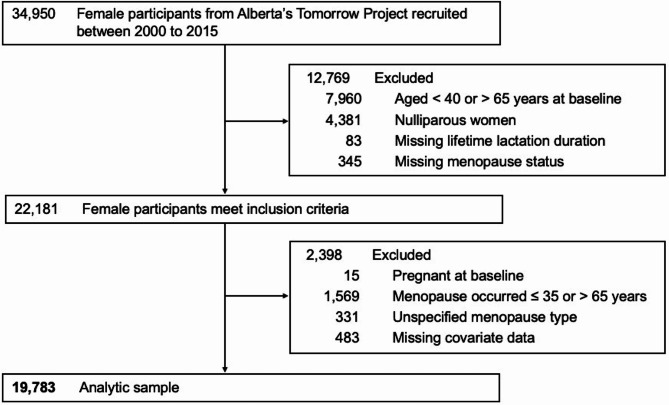
Flow diagram of included female participants from Alberta’s Tomorrow Project

We included parous females aged 40–65 years at baseline who provided data on lifetime lactation duration and menopausal status, excluding those who were pregnant at baseline, reported an extreme age at menopause (≤ 35 or > 65 years) or unspecified menopause type, or were missing covariate data (Fig. 1).

### Measures

Lifetime lactation duration, defined as the total number of months spent breastfeeding across all live births, was self-reported retrospectively at baseline and categorized into: not at all or < 1 month (reference group), 1–3 months, 4–6 months, 7–12 months, and ≥ 13 months.

Menopause characteristics were measured at baseline and each follow-up through self-report of final menstrual period, hysterectomy, or oophorectomy. We defined menopause type as: premenopause; natural menopause from a final menstrual period with no medical cause; surgical menopause induced through bilateral oophorectomy; indeterminate menopause from a final menstrual period induced through hysterectomy, whereby loss of menstruation with ovarian preservation renders the clinical timing of menopause inconclusive. We defined timing of natural menopause as age at one full year after the final menstrual period; and surgical and indeterminate menopause as age at surgery.

To estimate the total effect of lifetime lactation on timing of menopause, covariates were selected based on known or suspected associations with lactation duration [26–28] and timing of menopause (including gynecologic indications for iatrogenic menopause) [29–33], based on dataset availability, and using measurements at baseline and thus as proximal to the childbearing years as possible to represent potential confounding variables as opposed to mediating (pathway) variables. Demographic characteristics were participant year of birth and highest level of education. Health-related factors were parity, lifetime duration of hormonal contraceptive use, past or current smoking, body mass index (BMI), and physician-diagnosed diabetes, cardiovascular disease (including hypertension), and autoimmune disease. Menopausal hormone therapy (MHT) was measured at baseline and most follow-up contacts and defined as lifetime use and timing of initiation relative to menopause.

### Statistical analysis

We analyzed the association between lifetime lactation duration and timing of menopause using flexible parametric survival analysis. We modelled menopause type-specific hazards to account for competing risks given that menopause can only occur from one cause [34]. With age as the underlying time scale, person-time at risk was counted in years from age 35 to age at menopause or censoring due to the earliest of end of study follow-up, attrition, occurrence of a competing menopause type, or reaching either age 65 for natural menopause or age 60 for surgical or indeterminate menopause (owing to small event counts thereafter). We allowed this association to vary over time using restricted cubic splines with 4 degrees of freedom (3 internal knots) for the baseline hazard and 1 degree of freedom (no internal knots) for the effect of lactation. First, we estimated cumulative incidence functions with simulation-based 95% confidence intervals (CIs) [35]. Next, we estimated hazard ratios (HRs) and 95% CIs for earlier menopause, unadjusted and then adjusted for birth year, education, parity, duration of hormonal contraceptive use, and smoking to represent the total effect of lactation on menopause (i.e., controlling for confounding but inclusive of potential mediating pathways). An HR greater than 1 indicated earlier menopause in the group of interest (1–3, 4–6, 7–12, or ≥ 13 months) compared to the reference group (< 1 month) among those who were still premenopausal up to a given time point. To aid with interpretation of natural menopause HRs, we calculated the predicted age at natural menopause for participants in each lactation group using the adjusted survival model and plotted these values; this approach was not feasible for surgical or indeterminate menopause due to heavy censoring of natural menopause as a competing risk.

Among women who experienced menopause between 35 and 65 years, we analyzed the association between lifetime lactation duration and type of menopause using multinomial logistic regression, with natural menopause as the reference outcome group. We estimated odds ratios (ORs) and 95% CIs, unadjusted and adjusted for birth year, education, parity, duration of hormonal contraceptive use, and smoking.

We conducted five sensitivity analyses. First, we explored whether associations differed when restricted to women with 2 births (the largest parity group) to distinguish the effects of lactation from that of underlying number of births. Second, we accounted for the potential influence of MHT use prior to menopause by adding premenopausal MHT as a censoring event for the survival models and excluding women reporting premenopausal MHT from the multinomial models. Third, we further adjusted for baseline BMI and chronic medical conditions, which may have represented potential mediators (i.e., causal pathway variables that are influenced by lactation and in turn influence menopause timing) instead of confounders in the associations studied depending on each woman’s health trajectory and age at enrollment. Fourth, we explored potential reverse causation, wherein a shorter reproductive window given early onset of menopause could have systematically reduced lifetime lactation duration, by restricting to women who experienced menopause > 40 years, at which point most parous women have completed childbearing [36]. Fifth, for survival models only, we accounted for potential informative censoring using stabilized inverse probability of censoring weights estimated using exposure and covariate data [37, 38].

Data were cleaned in Stata MP version 17 and analyzed and visualized in R version 4.2.2 [39, 40].

## Results

We analyzed 19,783 females aged 40–65 at ATP Study baseline (Fig. 1). For lifetime lactation duration, 19.8% of women reported < 1 month, 12.1% reported 1–3 months, 11.7% reported 4–6 months, 18.8% reported 7–12 months, and 37.7% reported ≥ 13 months. Demographic and health gradients across lifetime lactation were evident for most characteristics (Table 1). A later year of birth, university or post-graduate degree, higher order parity, and never smoking were more frequent in longer lactation groups. Conversely, mean BMI was lower and chronic medical conditions and MHT were less frequent in longer lactation groups.

**Table 1:** Baseline characteristics of Alberta’s Tomorrow Project female participants by lifetime lactation duration

Characteristic	Lifetime Lactation Length									
	< 1 month *N* = 3,911		1–3 months *N* = 2,386		4–6 months *N* = 2,307		7–12 months *N* = 3,716		≥ 13 months *N* = 7,463	
	*n*	%	*n*	%	*n*	%	*n*	%	*n*	%
Birth year										
1930s	97	2.5	59	2.5	35	1.5	34	0.9	52	0.7
1940s	1403	35.9	628	26.3	528	22.9	553	14.9	759	10.2
1950s	1711	43.7	1068	44.8	1093	47.4	1779	47.9	3494	46.8
1960s	666	17.0	568	23.8	587	25.4	1207	32.5	2730	36.6
1970s	34	0.9	63	2.6	64	2.8	143	3.8	428	5.7
Age at baseline, mean (SD)	54.2	(6.7)	52.9	(6.9)	52.8	(7.0)	51.6	(6.7)	50.7	(6.6)
Education										
High school or less	2275	58.2	1125	47.2	949	41.1	1249	33.6	2013	27.0
College degree	1062	27.2	766	32.1	730	31.6	1212	32.6	2143	28.7
University degree	431	11.0	371	15.5	456	19.8	942	25.3	2505	33.6
Post-graduate degree	143	3.7	124	5.2	172	7.5	313	8.4	802	10.7
Parity										
1	884	22.6	622	26.1	505	21.9	501	13.5	275	3.7
2	1936	49.5	1115	46.7	1231	53.4	2182	58.7	3231	43.3
≥ 3	1091	27.9	649	27.2	571	24.8	1033	27.8	3957	53.0
Smoking status										
Never	1484	37.9	944	39.6	1039	45.0	1903	51.2	4418	59.2
Former	1661	42.5	1009	42.3	922	40.0	1437	38.7	2514	33.7
Current	766	19.6	433	18.1	346	15.0	376	10.1	531	7.1
Hormonal contraceptive use										
Never used	312	8.0	204	8.5	194	8.4	281	7.6	702	9.4
Ever used	3599	92.0	2182	91.5	2113	91.6	3435	92.4	6761	90.6
Years of use, mean (SD)	6.3	(4.7)	6.8	(5.3)	7.0	(5.3)	7.5	(5.5)	7.1	(5.4)
Body mass index, mean (SD)	28.3	(6.2)	27.9	(6.2)	27.2	(5.6)	26.9	(5.6)	26.3	(5.4)
Chronic medical conditions										
Diabetes	251	6.5	140	5.9	110	4.8	187	5.1	332	4.5
Cardiovascular disease	1280	32.8	673	28.3	556	24.1	803	21.7	1339	18.0
Autoimmune	318	8.2	212	9.0	184	8.0	326	8.8	590	7.9
Menopausal hormone therapy										
Never used	2111	54.0	1383	58.0	1399	60.7	2436	65.6	5287	70.9
Ever used	1798	46.0	1000	42.0	907	39.3	1280	34.4	2174	29.1
Premenopausal initiation	628	34.9	362	36.2	361	39.8	516	40.3	910	41.9
Postmenopausal initiation	814	45.3	448	44.8	414	45.6	608	47.5	1067	49.1
Initiation timing unknown	356	19.8	190	19.0	132	14.6	156	12.2	197	9.1

SD: standard deviation

**Fig. 2:**
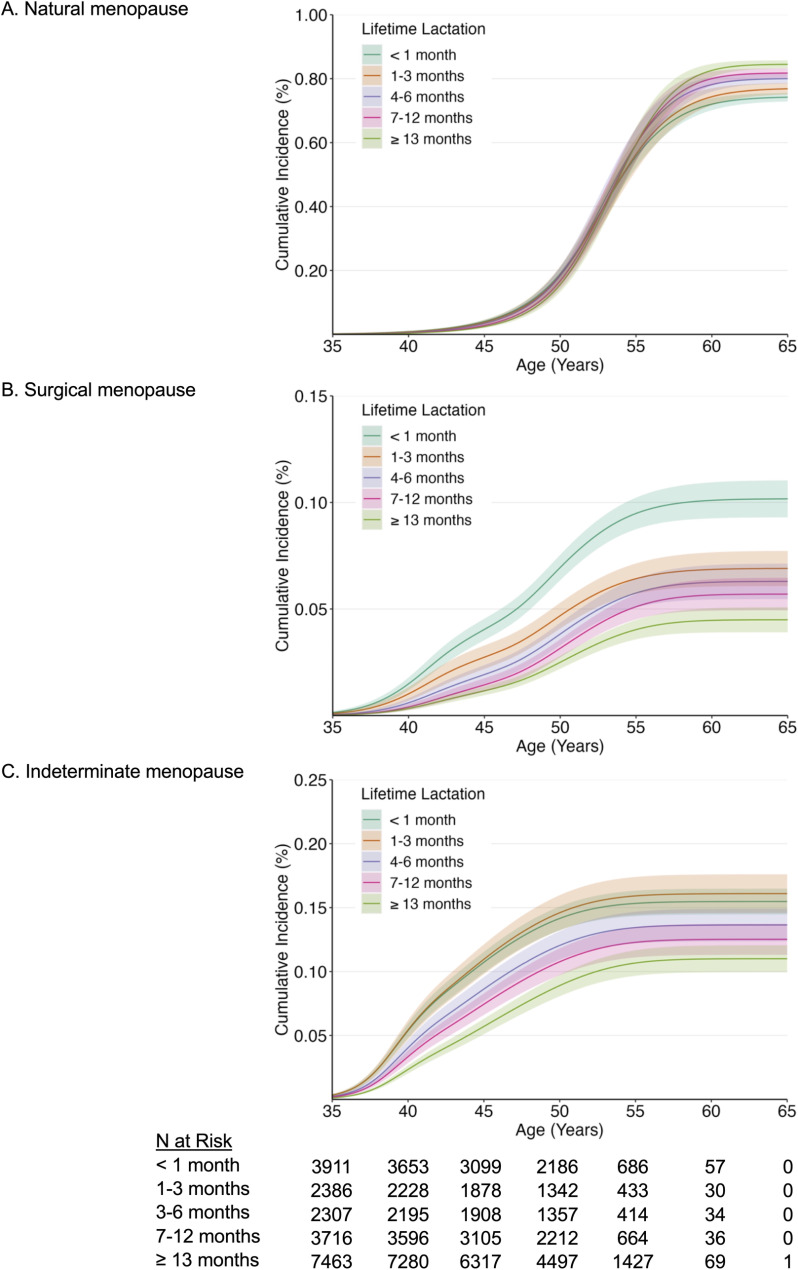
Cumulative incidence of natural, surgical, and indeterminate menopause by lifetime lactation. Y-axes of panel B and C were re-sized for better visualization. The number of participants at risk at each time point excludes participants who experienced menopause and participants who were censored before menopause

By study end, 74.8% of women experienced menopause; natural menopause was most common (57.4%), followed by indeterminate (12.1%) and surgical menopause (5.4%). For age at natural menopause, there was a positive gradient with higher cumulative incidence curves among longer lactation groups and noticeable separation amongst curves beginning at age 55 (Fig. 2). Conversely, for age at surgical and indeterminate menopause, there was a negative gradient with lower cumulative incidence curves among longer lactation groups and noticeable separation amongst curves across nearly all ages (Fig. 2).

**Fig. 3:**
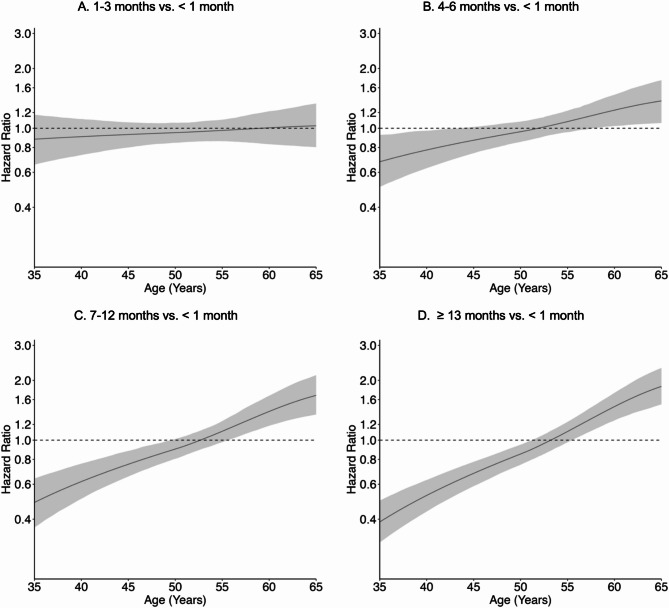
Adjusted association of lifetime lactation and timing of natural menopause. Models controlled for birth year, education, parity, duration of hormonal contraceptive use, and smoking

**Fig. 4:**
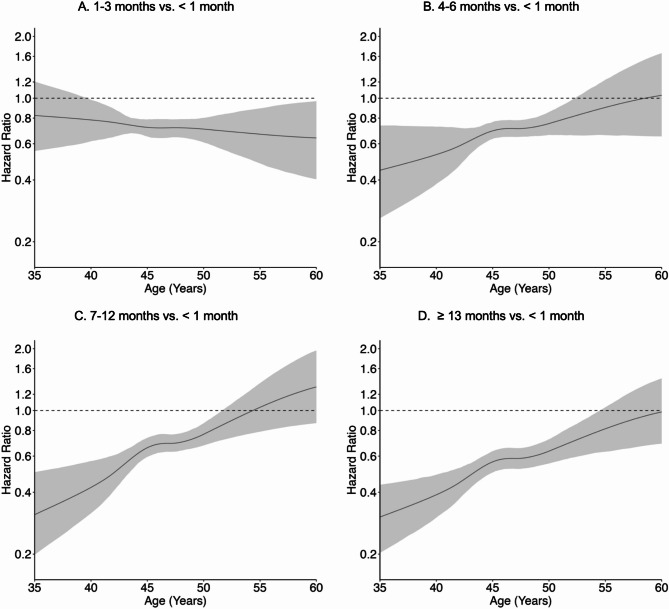
Adjusted association of lifetime lactation and timing of surgical menopause. Models controlled for birth year, education, parity, duration of hormonal contraceptive use, and smoking

**Fig. 5:**
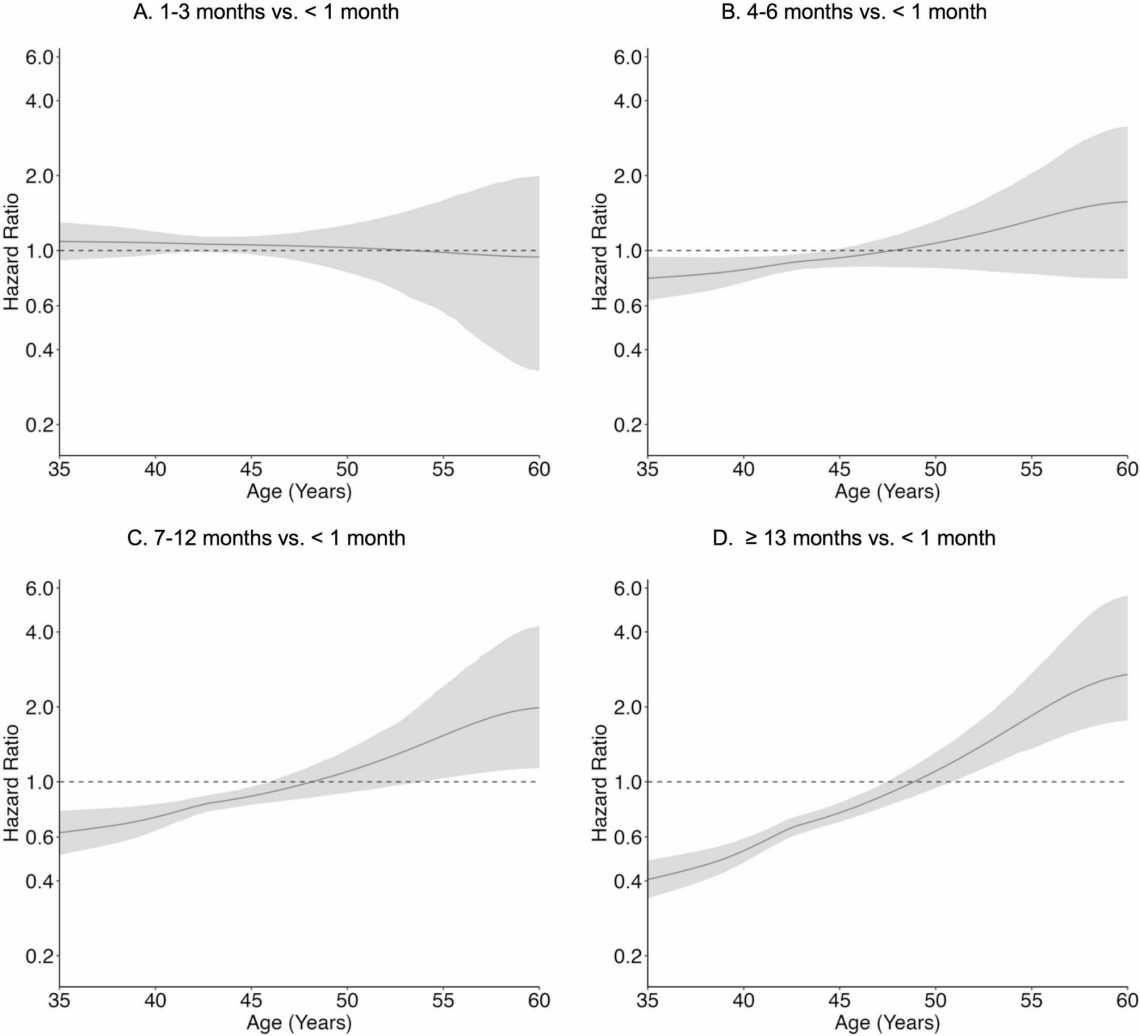
Adjusted association of lifetime lactation and timing of indeterminate menopause (premenopausal hysterectomy). Models controlled for birth year, education, parity, duration of hormonal contraceptive use, and smoking

Longer lifetime lactation was associated with timing of natural, surgical, and indeterminate menopause in crude and adjusted models; associations were dose-response in nature, reflected by increasing magnitude with longer lactation, and varied over time based on underlying age. For natural menopause (Fig. 3, Supplemental Table S1), adjusted HR curves displayed a positive slope beginning at 4–6 months of lactation that traversed the null around ages 50 to 55 and steepened with longer lifetime lactation. For example, adjusted HRs for ≥ 13 months of lactation indicated that risk of natural menopause was lower between ages 35 to 50 (age 45: 0.68, 95% CI 0.59–0.78), similar between ages 50 to 55 (age 55: 1.11, 95% CI 0.98–1.24), and elevated between ages 55 to 65 (age 60: 1.48, 95% CI 1.26–1.73), compared to < 1 month of lactation. The distributions of predicted mean age at natural menopause from the adjusted survival model were indeed centered at slightly older ages and had less variability with longer lifetime lactation (Supplemental Figure S1). For surgical menopause (Fig. 4, Supplemental Table S1), the adjusted HR curve at 1–3 months of lactation was fairly flat and showed a protective effect (age 50: 0.71, 95% CI 0.61–0.80), whereas the adjusted HR curves from 4 to 6 months of lactation onward displayed a positive slope that enclosed the null around age 52 and steepened with longer lifetime lactation. Adjusted HRs for ≥ 13 months of lactation indicated that risk of surgical menopause was lower risk between ages 35 to 55 (age 45: 0.56, 95% CI 0.50–0.63) and similar between ages 55 to 60 (age 55: 0.82, 95% CI 0.63–1.04), compared to < 1 month of lactation. For indeterminate menopause (Fig. 5, Supplemental Table S1), the adjusted HR curve at 1–3 months of lactation was fairly flat and showed a null effect (age 50: 1.03, 95% CI 0.82–1.27), whereas the adjusted HR curves from 4 to 6 months of lactation onward displayed a positive slope that traversed the null around age 50 and steepened with longer lifetime lactation. Adjusted HRs for ≥ 13 months of lactation indicated that risk of indeterminate menopause was lower between ages 35 to 50 (age 45: 0.75, 95% CI 0.69–0.82) and elevated between ages 50 to 60 (age 55: 1.84, 95% CI 1.36–2.72), compared to < 1 month of lactation. However, 95% CI width increased rapidly after age 50.

Among women who experienced menopause, lifetime lactation had an inverse and dose-response association with surgical and indeterminate menopause in crude and adjusted models using natural menopause as the reference outcome group (Table 2). For example, ≥ 13 months of lactation was associated with lower odds of surgical (adjusted OR 0.54, 95% CI 0.45–0.66) and indeterminate menopause (adjusted OR 0.63, 95% CI 0.55–0.73) compared to < 1 month of lactation.

**Table 2:** Association of lifetime lactation and menopause type among women who experienced menopause

Lifetime Lactation (months)	No. (%) with the outcome						Odds Ratio 95% CI							
	Natural (ref)		Surgical		Indeterminate		Surgical				Indeterminate			
							Crude		Adjusted		Crude		Adjusted	
< 1 (Ref)	2281	(70.9)	357	(11.1)	580	(18.0)	1	–	1	–	1	–	1	–
1–3	1362	(72.9)	144	(7.7)	362	(19.4)	0.68	(0.55–0.83)	0.73	(0.60–0.90)	1.05	(0.90–1.21)	1.03	(0.89–1.20)
4–6	1385	(76.8)	124	(6.9)	294	(16.3)	0.57	(0.46–0.71)	0.66	(0.53–0.82)	0.83	(0.71–0.98)	0.85	(0.73-1.00)
7–12	2128	(78.1)	172	(6.3)	426	(15.6)	0.52	(0.43–0.63)	0.64	(0.53–0.79)	0.79	(0.69–0.90)	0.78	(0.68–0.91)
≥ 13	4197	(80.9)	266	(5.1)	725	(14.0)	0.40	(0.34–0.48)	0.54	(0.45–0.66)	0.68	(0.60–0.77)	0.63	(0.55–0.73)

Adjusted models controlled for birth year, education, parity, duration of hormonal contraceptive use, and smoking

Results from the survival (Supplemental Figures S2, S3, S4) and multinomial logistic (Supplemental Table S2) models were robust to sensitivity analyses restricting to women with 2 births, censoring or excluding women at initiation of premenopausal MHT, additionally adjusting for baseline BMI and chronic medical conditions, restricting to women with menopause > 40 years, and inverse probability of censoring weighting. Of note, restricting to women with menopause > 40 years attenuated point estimates and decreased precision for the associations between lifetime lactation and indeterminate menopause.

## Discussion

This longitudinal cohort study detected a dose-response and time-dependent relationship between lifetime lactation and menopausal characteristics. Longer lactation was associated with a slight narrowing of the distribution for timing of natural menopause centered around 50 to 55 years. Risk of surgical menopause through bilateral oophorectomy decreased with increasing lactation, particularly before age 55. Longer lactation was also associated with decreased risk of indeterminate menopause through premenopausal hysterectomy with ovarian preservation before age 50 but increased risk of indeterminate menopause thereafter; however, this association was attenuated in sensitivity analysis restricting to menopause after 40 years and may thus have been partly induced through reverse causation. Findings suggest that optimizing the timing of natural menopause to the biological norm of 50 to 55 years and reducing the risk of early surgical and indeterminate menopause may be novel maternal benefits associated with breastfeeding.

Our finding of a time-dependent effect of lactation on natural menopause aligns with existing evidence. An analysis of 2,377 parous women in the Study of Women’s Health Across the Nation found no association between lactation duration and timing of natural menopause after controlling for social factors [21], however the study’s selection criteria systematically excluded women with early natural menopause (i.e., < 45 years) [41]. Conversely, an analysis of 59,388 parous women in the Nurses’ Health Study II focused solely on the outcome of early natural menopause and reported that longer lactation was protective [20].

Our work, in conjunction with prior studies [20, 42, 43], do not support the follicle sparing hypothesis as the sole mechanism between lactation and natural menopause; there is evidence of a dose-response effect of lactation, but it does not appear unidirectional towards delayed menopause. Rather, our HR curves displayed an intriguing trend wherein longer lactation was associated with a slight narrowing of the distribution for timing of menopause centered around 50 to 55 years. The population mean age of natural menopause at 50 to 53 years is fairly consistent across geographies and generations [7, 44], suggesting a biological norm in the rate of follicular decline underpinned perhaps by genetics or evolution [45–47]. This biological norm is also reflected in epidemiologic data showing that early natural menopause (< 45 years) has numerous potential harms, but also that late natural menopause (> 55 years) bears risks for breast, uterine, ovarian cancer [29]. It is possible that homeostatic mechanisms exist in the ovaries to permit follicle sparing up to this biological norm, but not thereafter. This would explain the diminishment of effect we observed for lactation around age 50 for natural menopause, which has been similarly observed in studies of other follicle sparing exposures such as parity where effects appear exclusively in relation to early menopause [20, 21, 42, 48]. Future biomedical research is needed to elucidate the precise physiologic impacts of lactation intensity and duration on ovarian activity and explore a potential point of diminished returns on follicle sparing activity. In terms of increased onset of menopause after age 50, broader positive health effects of lactation may play a role. Obesity is one of the few known risk factors for late menopause [49, 50] and is less common in women who have longer breastfeeding duration as lactation is bidirectionally associated with lower adiposity [51, 52]. Our findings persisted after controlling for baseline BMI, but residual confounding or mediating effects are possible without longitudinal measurement spanning back to preconception.

To our knowledge, our analysis of lactation in relation to iatrogenic menopause is a novel addition to the literature. Our finding of decreased risks of surgical menopause and premenopausal hysterectomy before age 50 with longer lactation is consistent with evidence showing that lactation is associated with reduced incidence and symptom severity of uterine fibroids and endometriosis [22–24, 53, 54], which are indicated in approximately 20% of bilateral oophorectomies and 60% of hysterectomies in early adulthood [55–57]. Importantly, these data suggest a novel sequelae of lactation-related health benefits for women through reducing early oophorectomy and hysterectomy and their downstream risks of cardiovascular disease and dementia [58–60].

Our finding of increased risk of premenopausal hysterectomy after age 50, albeit partly explained by possible reverse causation, was unexpected and hypothesis-generating on the potential role of lactation in common indications for hysterectomy without oophorectomy after 50 years of age. For example, pelvic organ prolapse is indicated in up to 50% of hysterectomies performed at age 50 or older [55, 57, 61], and is an anatomic condition arising from deterioration of pelvic floor muscles and ligaments through pregnancy, birth, aging, and other load-bearing exposures [62]. One possible interpretation is that breastfeeding may interfere with tissue remodelling through supressing estrogen and its regenerative effects on pelvic floor connective tissue [63]. This theoretical mechanism has received limited attention in population research, with only two clinical studies reporting no evidence of an association between breastfeeding and pelvic floor dysfunction though limited by lack of statistical power [64, 65]. Another possible interpretation involves systematic differences in mode of delivery across lactation groups. Compared to Cesarean birth, vaginal birth is associated with fewer breastfeeding difficulties and longer breastfeeding duration [66, 67], but is also a causal factor for pelvic organ prolapse as women age [32]. Future research should revisit the association of lactation and hysterectomy in conjunction with data on clinical indication for this procedure, and explore the underlying roles (if any) of pelvic floor disorders and mode of delivery.

This study has limitations to consider. Data were measured through self-report, with retrospective collection of lactation data and a combination of retrospective and prospection collection of menopause data depending on women’s ages at enrollment. Recall of lactation duration and menopause characteristics generally exhibits moderate-to-high accuracy up to 20 years later [68–73], yet the possibility of memory error and thus random misclassification bias cannot be ruled out. Residual confounding is probable given baseline measurement of covariates in midlife, as some characteristics (e.g., education, BMI) are often dynamic and may have differed during the childbearing years. Temporal and cultural changes in breastfeeding determinants, hysterectomy patterns, and opportunistic oophorectomy recommendations are well-documented; [74–76] given that we lacked data on the calendar years a women breastfed and, where applicable, underwent gynecologic procedures, analysis of the impact of historical trends in women’s health on reproductive associations could be a valuable area of future inquiry. The ATP Study is largely representative of the Alberta female population; however, unintentionally low sampling from diverse race/ethnic groups and individuals with post-secondary education places some limits on the external validity of results [25].

## Conclusion

This cohort study found a dose-response relationship between lifetime lactation and menopause characteristics that is dynamic over the continuum of aging. Longer lactation was associated with delayed onset of natural menopause before age 50 and quicker onset of natural menopause after age 55, thus concentrating natural menopause during the biological norm of 50 to 55 years. Longer lactation was also associated with reduced risks of early surgical menopause (bilateral oophorectomy) and indeterminate menopause (premenopausal hysterectomy with ovarian preservation) before age 50, with possible evidence of slightly increased risk of indeterminate menopause after age 50. Additional studies are needed to better understand how lactation and associated obstetric and health factors may impact gynecologic physiology, conditions, and surgery rates as women age. Findings suggest that scaling up healthcare policies and practices shown to improve population breastfeeding rates could help optimize the type and timing of menopause women experience in midlife.

## Electronic supplementary material

Below is the link to the electronic supplementary material.

Supplementary Material 1

## Data Availability

Requests to access the data used in this study can be directed to the Alberta’s Tomorrow Project team at ATP.Research@albertahealthservices.ca.

## Abbreviations

ATP: Alberta’s Tomorrow Project
BMI: Body mass index
CI: Confidence interval
HR: Hazard ratio
MHT: Menopausal hormone therapy
