# Supplementary material for: Association of lifetime lactation and characteristics of menopause: a longitudinal cohort study
Source: BMC Public Health. 2024 Nov 11;24:3112. doi: 10.1186/s12889-024-20508-7 (PMC11552320; doi:10.1186/s12889-024-20508-7)
Supplement: Supplementary file 1 — Supplementary Material 1 [file 12889_2024_20508_MOESM1_ESM.docx]

**Supplementary Material**

**Association of lifetime lactation with characteristics of menopause: A longitudinal cohort study**

Supplemental Table S1. Association of lifetime lactation and timing of natural, surgical, and indeterminate menopause: Summary of hazard ratios

Supplemental Table S2. Sensitivity analyses of the adjusted association between lifetime lactation and menopause type among women who experienced menopause

Supplemental Figure S1. Predicted distribution of age at natural menopause from adjusted survival models by lifetime lactation group

Supplemental Figure S2. Sensitivity analyses of the adjusted association between lifetime lactation and timing of natural menopause

Supplemental Figure S3. Sensitivity analyses of the adjusted association between lifetime lactation and timing of surgical menopause

Supplemental Figure S4. Sensitivity analyses of the adjusted association between lifetime lactation and timing of indeterminate menopause (premenopausal hysterectomy)

**Supplemental Table S1. Association of lifetime lactation and timing of natural, surgical, and indeterminate menopause: Summary of hazard ratios**

| Lifetime  Lactation  (months) | Hazard Ratio (95% Confidence Interval) | | | | | | | | | | | | | | | | |
| --- | --- | --- | --- | --- | --- | --- | --- | --- | --- | --- | --- | --- | --- | --- | --- | --- | --- |
|  | Crude | | | | | | | | | Adjusted | | | | | | | |
|  | 40 years | | | 45 years | | 50 years | | 55 years | | 40 years | | 45 years | | 50 years | | 55 years | |
| Natural Menopause | | | | | | | | | | | | | | | | | |
| < 1 (ref) | 1 | | – | 1 | – | 1 | – | 1 | – | 1 | – | 1 | – | 1 | – | 1 | – |
| 1-3 | 0.92 | | (0.74-1.13) | 0.94 | (0.81-1.09) | 0.96 | (0.86-1.08) | 0.98 | (0.87-1.11) | 0.91 | (0.73-1.11) | 0.93 | (0.80-1.07) | 0.95 | (0.85-1.07) | 0.98 | (0.86-1.10) |
| 4-6 | 0.78 | | (0.63-0.95) | 0.87 | (0.75-1.01) | 0.96 | (0.86-1.07) | 1.08 | (0.96-1.22) | 0.78 | (0.63-0.97) | 0.87 | (0.75-1.02) | 0.96 | (0.85-1.08) | 1.08 | (0.96-1.23) |
| 7-12 | 0.61 | | (0.50-0.74) | 0.74 | (0.64-0.86) | 0.88 | (0.79-0.99) | 1.08 | (0.95-1.24) | 0.62 | (0.51-0.76) | 0.76 | (0.66-0.88) | 0.90 | (0.81-1.01) | 1.11 | (0.98-1.25) |
| ≥ 13 | 0.50 | | (0.42-0.60) | 0.65 | (0.56-0.75) | 0.81 | (0.73-0.92) | 1.06 | (0.94-1.21) | 0.53 | (0.44-0.63) | 0.68 | (0.59-0.78) | 0.85 | (0.75-0.95) | 1.11 | (0.98-1.24) |
| Surgical Menopause | | | | | | | | | | | | | | | | | |
| < 1 (ref) | 1 | | – | 1 | – | 1 | – | 1 | – | 1 | – | 1 | – | 1 | – | 1 | – |
| 1-3 | 0.68 | | (0.54-0.87) | 0.65 | (0.59-0.70) | 0.64 | (0.56-0.72) | 0.61 | (0.41-0.83) | 0.78 | (0.62-0.98) | 0.72 | (0.65-0.79) | 0.71 | (0.61-0.80) | 0.67 | (0.48-0.89) |
| 4-6 | 0.43 | | (0.33-0.59) | 0.57 | (0.52-0.63) | 0.63 | (0.56-0.70) | 0.78 | (0.55-1.04) | 0.53 | (0.38-0.73) | 0.69 | (0.63-0.77) | 0.75 | (0.66-0.86) | 0.90 | (0.66-1.23) |
| 7-12 | 0.31 | | (0.23-0.42) | 0.48 | (0.43-0.53) | 0.56 | (0.51-0.63) | 0.80 | (0.60-1.06) | 0.42 | (0.32-0.57) | 0.66 | (0.59-0.74) | 0.77 | (0.68-0.87) | 1.04 | (0.79-1.36) |
| ≥ 13 | 0.25 | | (0.20-0.31) | 0.36 | (0.33-0.40) | 0.42 | (0.38-0.47) | 0.56 | (0.44-0.73) | 0.39 | (0.30-0.49) | 0.56 | (0.50-0.63) | 0.63 | (0.55-0.73) | 0.82 | (0.63-1.04) |
| Indeterminate Menopause | | | | | | | | | | | | | | | | | |
| < 1 (ref) | 1 | – | | 1 | – | 1 | – | 1 | – | 1 | – | 1 | – | 1 | – | 1 | – |
| 1-3 | 1.03 | (0.92-1.14) | | 1.01 | (0.93-1.10) | 0.99 | (0.80-1.23) | 0.95 | (0.54-1.61) | 1.07 | (0.96-1.19) | 1.05 | (0.97-1.14) | 1.03 | (0.82-1.27) | 0.98 | (0.56-1.59) |
| 4-6 | 0.78 | (0.69-0.87) | | 0.87 | (0.80-0.94) | 0.99 | (0.80-1.20) | 1.22 | (0.77-1.86) | 0.84 | (0.75-0.94) | 0.94 | (0.86-1.02) | 1.07 | (0.85-1.32) | 1.32 | (0.81-2.05) |
| 7-12 | 0.65 | (0.58-0.73) | | 0.79 | (0.73-0.86) | 0.99 | (0.82-1.21) | 1.38 | (0.95-2.15) | 0.72 | (0.64-0.81) | 0.88 | (0.81-0.96) | 1.10 | (0.91-1.35) | 1.53 | (1.03-2.42) |
| ≥ 13 | 0.48 | (0.43-0.54) | | 0.68 | (0.63-0.74) | 1.00 | (0.85-1.17) | 1.67 | (1.21-2.41) | 0.53 | (0.47-0.59) | 0.75 | (0.69-0.82) | 1.10 | (0.95-1.31) | 1.84 | (1.36-2.72) |

Adjusted models controlled for birth year, education, parity, duration of hormonal contraceptive use, and smoking.

**Supplemental Table S2. Sensitivity analyses of association between lifetime lactation and menopause type among women who experienced menopause**

| Sensitivity analysis | Lifetime Lactation | Adjusted Odds Ratio (95% Confidence Interval) | | | | | |
| --- | --- | --- | --- | --- | --- | --- | --- |
|  |  | Surgical | | | Indeterminate | | |
| 1. Restricted to parity of 2 (N = 9,695) | < 1 month (ref) | 1 | | - | 1 | | - |
|  | 1-3 months | 0.65 | (0.48-0.89) | | 0.90 | (0.72-1.12) | |
|  | 4-6 months | 0.67 | (0.50-0.91) | | 0.81 | (0.65-1.01) | |
|  | 7-12 months | 0.64 | (0.48-0.84) | | 0.77 | (0.63-0.94) | |
|  | ≥ 13 months | 0.53 | (0.40-0.71) | | 0.59 | (0.48-0.73) | |
| 2. Censored at initiation of premenopausal hormone therapy (N = 19,689) | < 1 month (ref) | 1 | | - | 1 | | - |
|  | 1-3 months | 0.74 | (0.59-0.93) | | 1.06 | (0.90-1.24) | |
|  | 4-6 months | 0.63 | (0.50-0.81) | | 0.85 | (0.72-1.01) | |
|  | 7-12 months | 0.63 | (0.51-0.79) | | 0.79 | (0.68-0.92) | |
|  | ≥ 13 months | 0.51 | (0.42-0.63) | | 0.61 | (0.53-0.71) | |
| 3. Additionally adjusted for body mass index and chronic medical conditions (N = 19,431) | < 1 month (ref) | 1 | | - | 1 | | - |
|  | 1-3 months | 0.75 | (0.61-0.93) | | 1.04 | (0.89-1.21) | |
|  | 4-6 months | 0.70 | (0.56-0.87) | | 0.85 | (0.72-1.00) | |
|  | 7-12 months | 0.68 | (0.55-0.84) | | 0.80 | (0.69-0.93) | |
|  | ≥ 13 months | 0.59 | (0.48-0.71) | | 0.65 | (0.57-0.75) | |
| 4. Restricted to menopause >40 years (N = 18,469) | < 1 month (ref) | 1 | - | | 1 | - | |
|  | 1-3 months | 0.72 | (0.57-0.91) | | 0.99 | (0.82-1.20) | |
|  | 4-6 months | 0.71 | (0.56-0.91) | | 0.86 | (0.71-1.05) | |
|  | 7-12 months | 0.72 | (0.58-0.90) | | 0.88 | (0.74-1.05) | |
|  | ≥ 13 months | 0.58 | (0.47-0.72) | | 0.73 | (0.62-0.87) | |

Models controlled for birth year, education, parity (not applicable for sensitivity analysis 1), duration of hormonal contraceptive use, and smoking. Chronic medical conditions were diabetes, cardiovascular disease, and autoimmune disease.

**Supplemental Figure S1. Predicted distribution of age at natural menopause from adjusted survival models by lifetime lactation group**


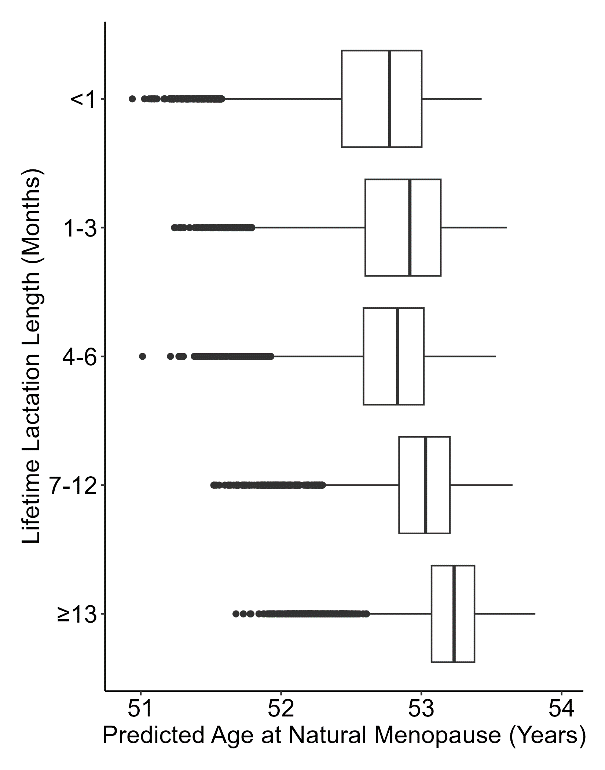


**Supplemental Figure S2. Sensitivity analyses of the adjusted association between lifetime lactation and timing of natural menopause**

|  | A. 1-3 months vs. < 1 month | B. 4-6 months vs. < 1 month | C. 7-12 months vs. < 1 month | D. ≥ 13 months vs. < 1 month |
| --- | --- | --- | --- | --- |
| 1. | 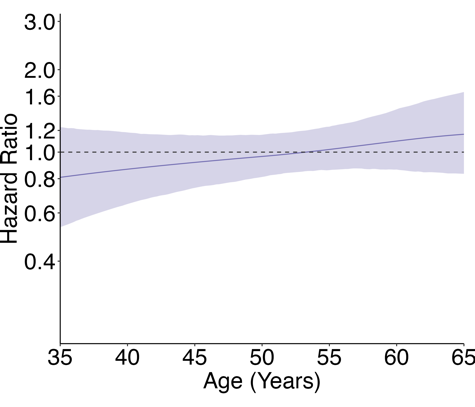 | 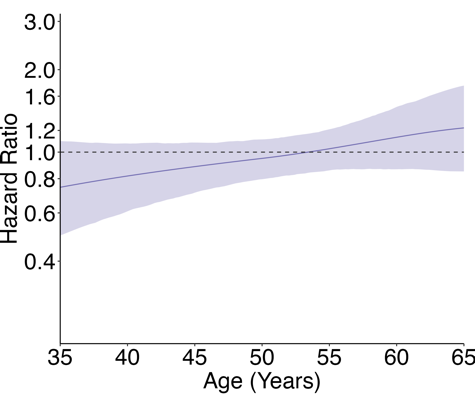 | 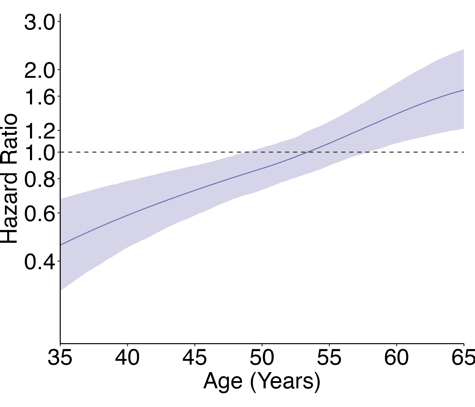 | 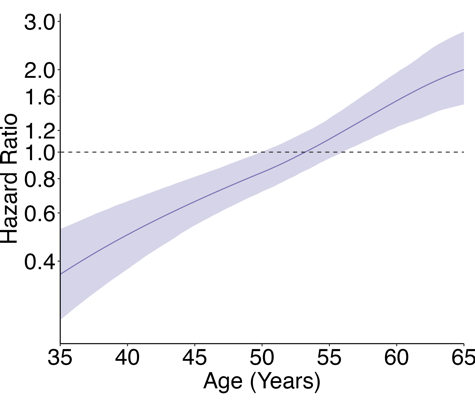 |
| 2. | 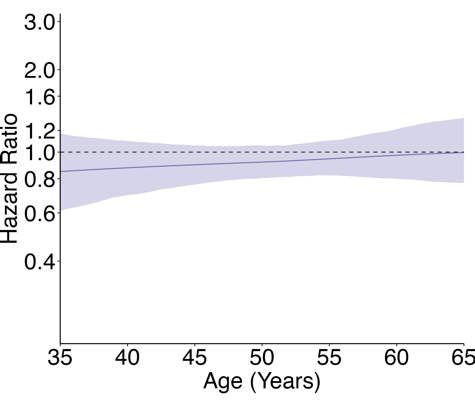 | 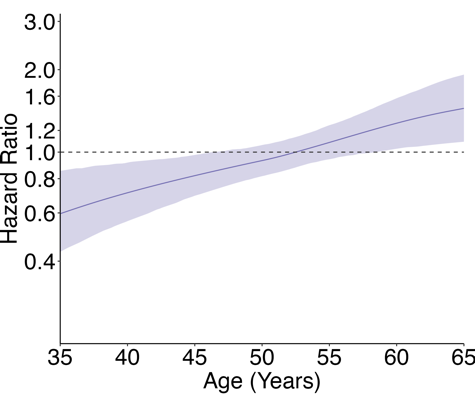 | 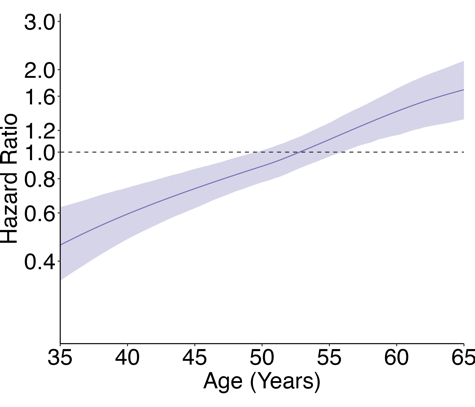 | 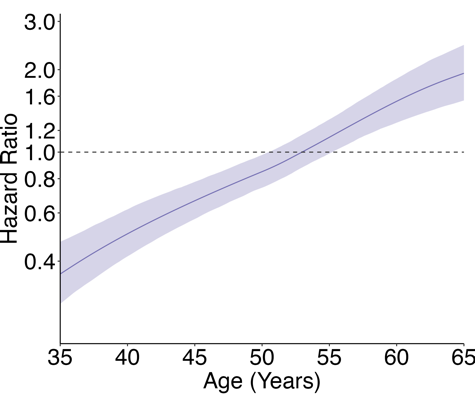 |
| 3. | 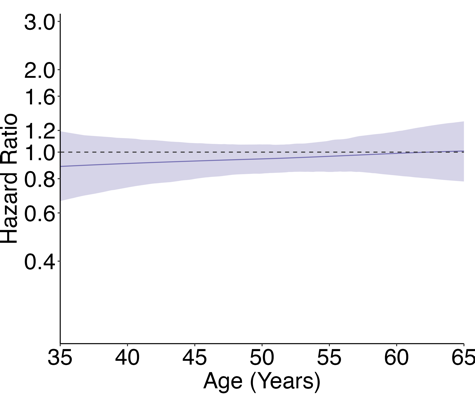 | 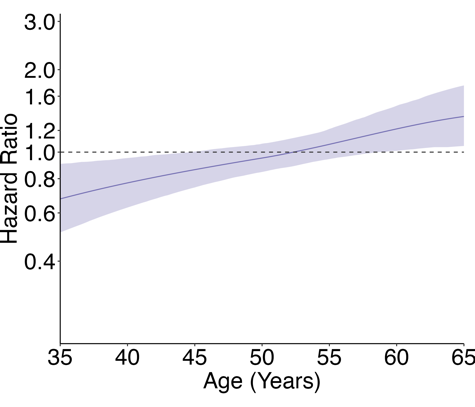 | 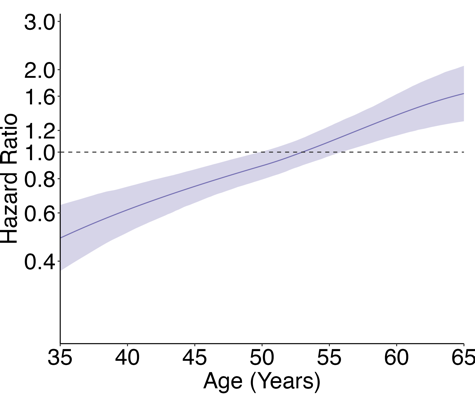 | 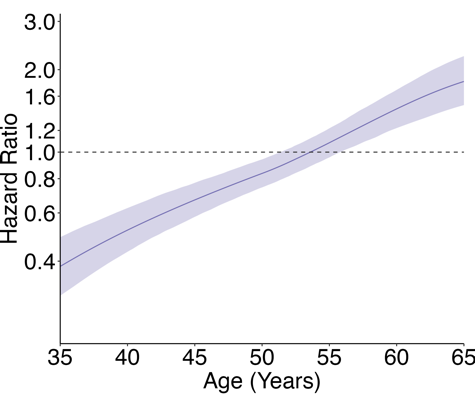 |
| 4. | 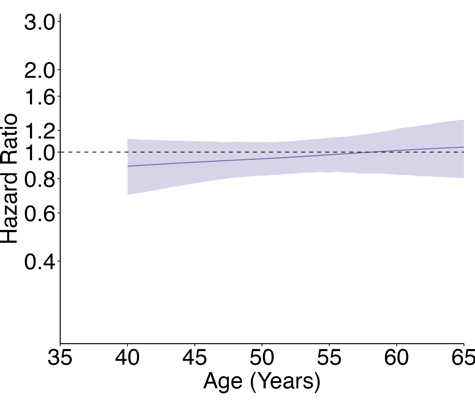 | 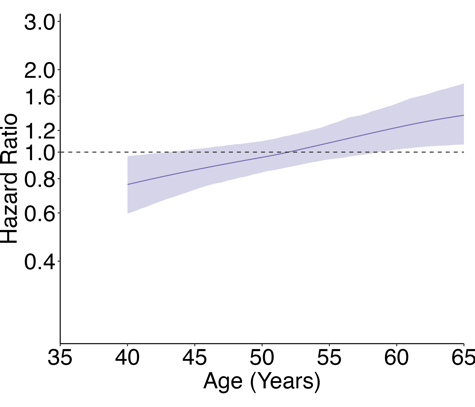 | 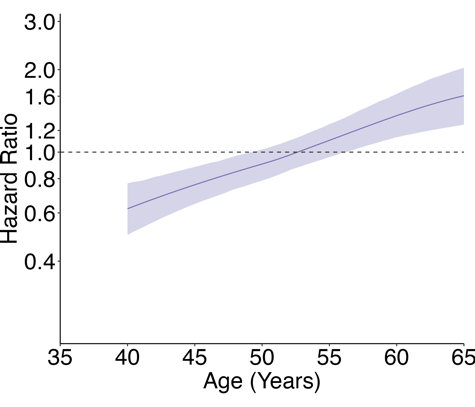 | 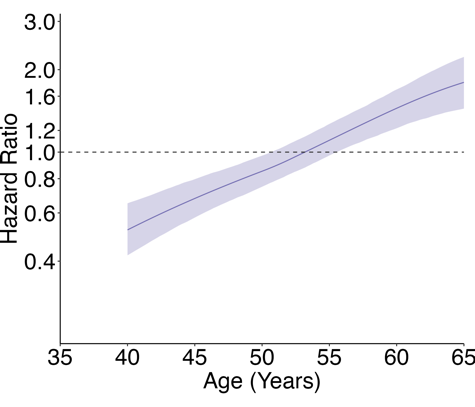 |
| 5. | 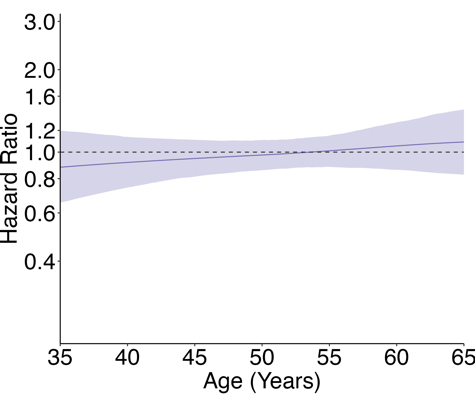 | 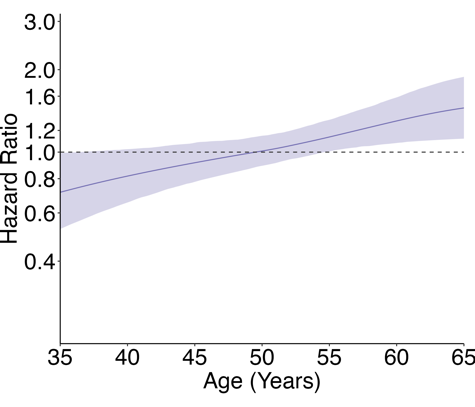 | 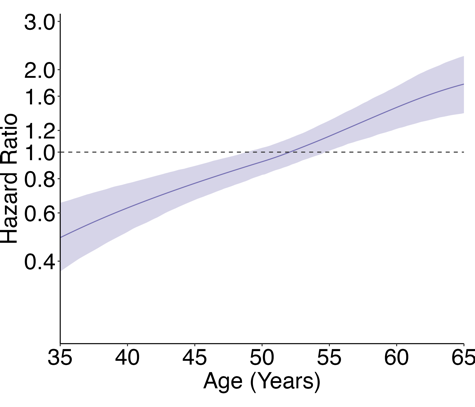 | 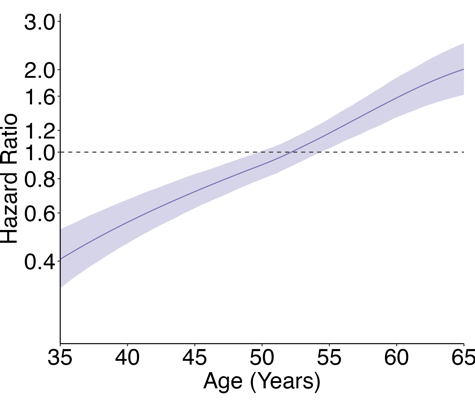 |

1. Restricted to parity of 2 (N = 9,695)

2. Censored at initiation of premenopausal hormone therapy (N = 19,689)

3. Additionally adjusted for body mass index and chronic medical conditions (diabetes, cardiovascular disease, autoimmune disease) (N = 19,431)

4. Restricted to menopause >40 years (N = 18,469)

5. Weighted by inverse probability of censoring weights (N = 19,417)

Models controlled for birth year, education, parity (not applicable for sensitivity analysis 1), duration of hormonal contraceptive use, and smoking.

Y-axes of all figures were restricted to the same range as the HR figures from the main model for better visual comparison.

**Supplemental Figure S3. Sensitivity analyses of the adjusted association between lifetime lactation and timing of surgical menopause**

|  | A. 1-3 months vs. < 1 month | B. 4-6 months vs. < 1 month | C. 7-12 months vs. < 1 month | D. ≥ 13 months vs. < 1 month |
| --- | --- | --- | --- | --- |
| 1. | 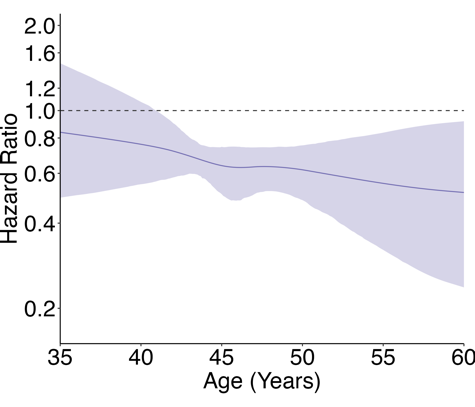 | 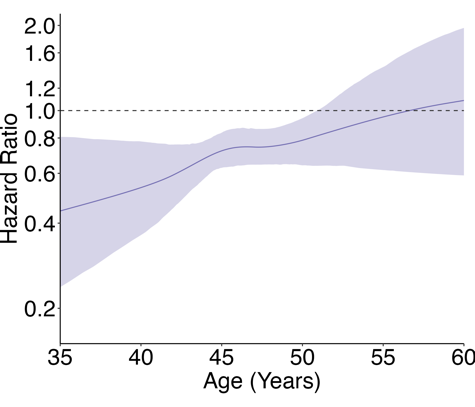 | 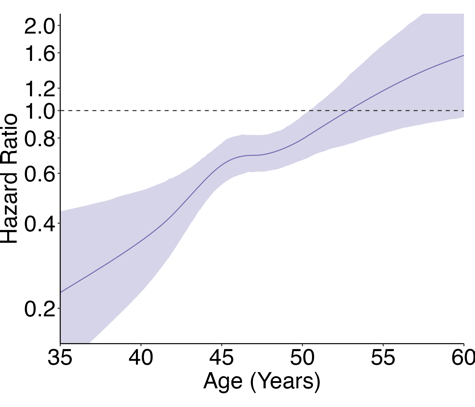 | 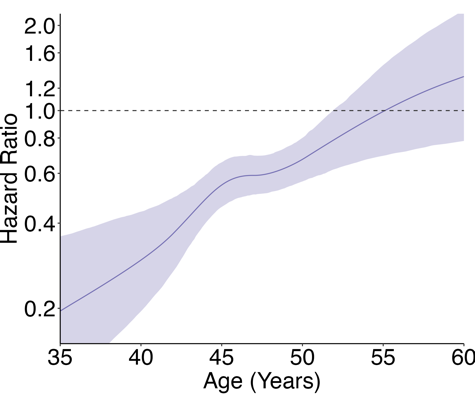 |
| 2. | 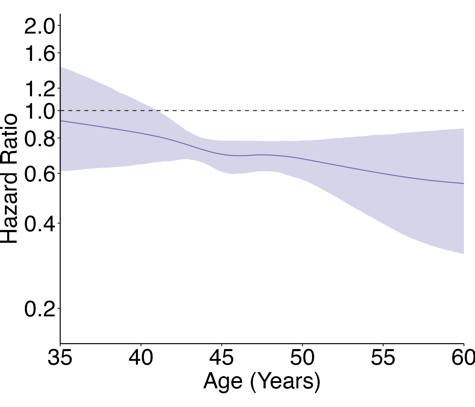 | 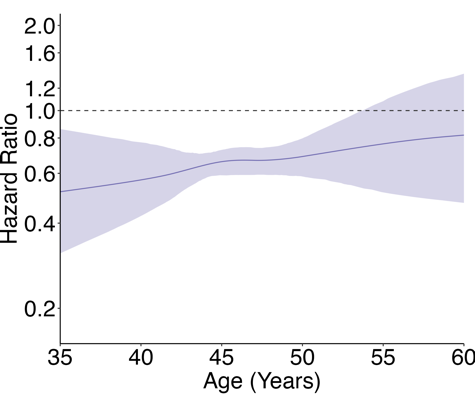 | 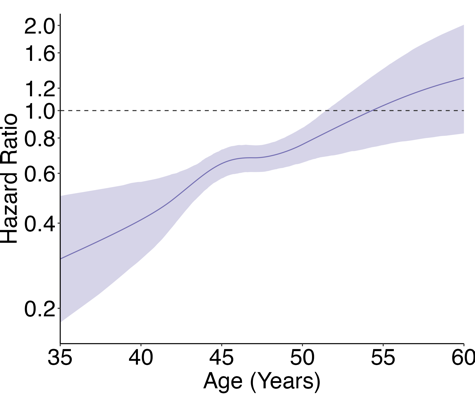 | 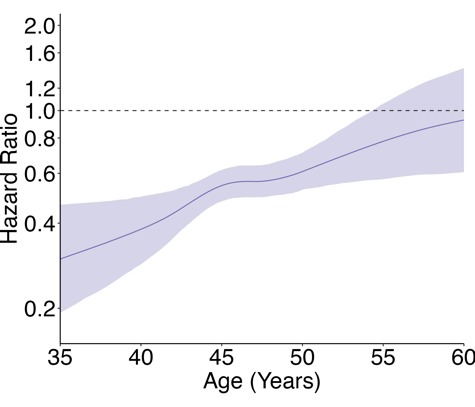 |
| 3. | 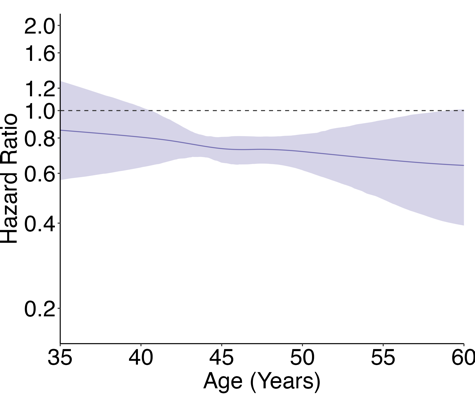 | 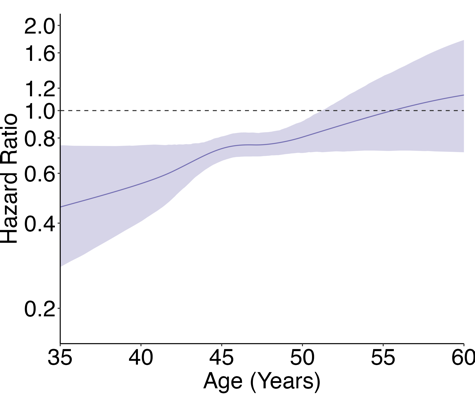 | 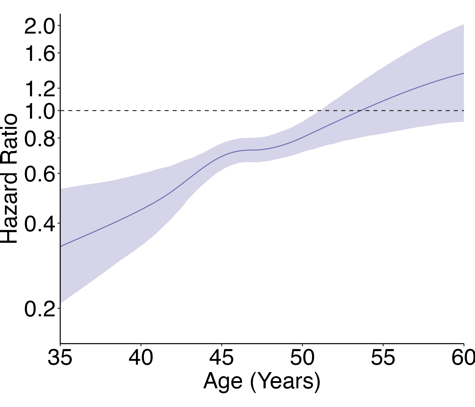 | 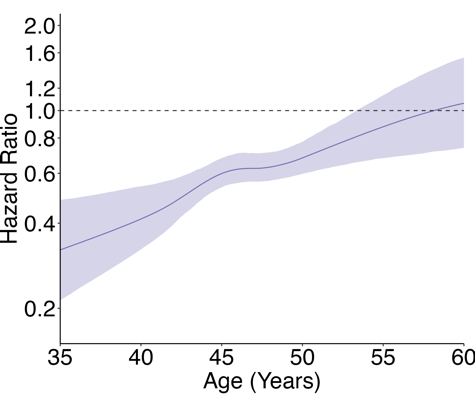 |
| 4. | 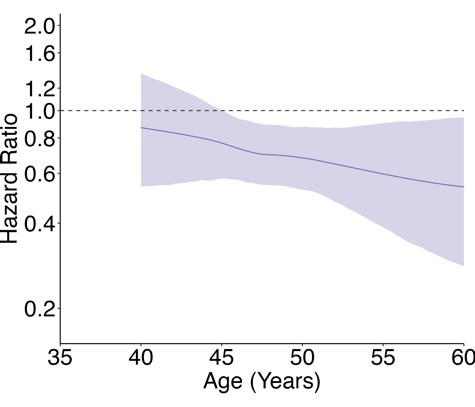 | 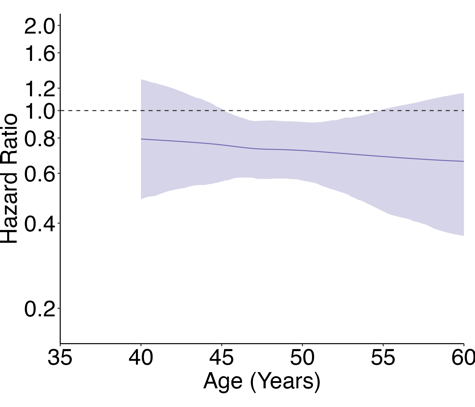 | 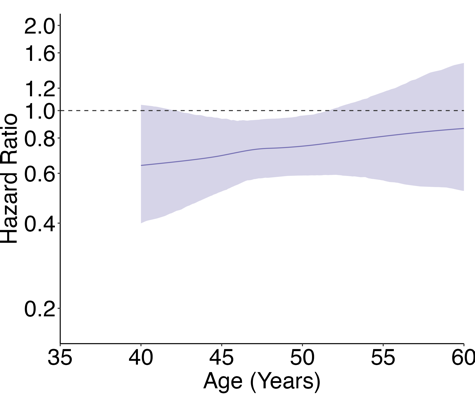 | 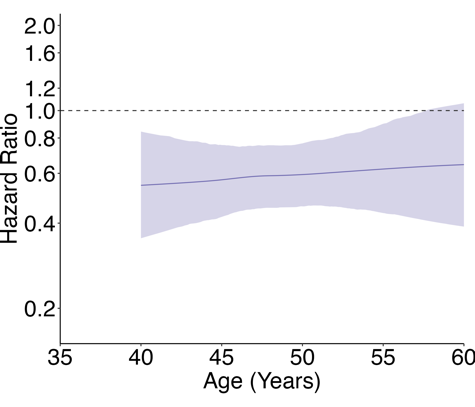 |
| 5. | 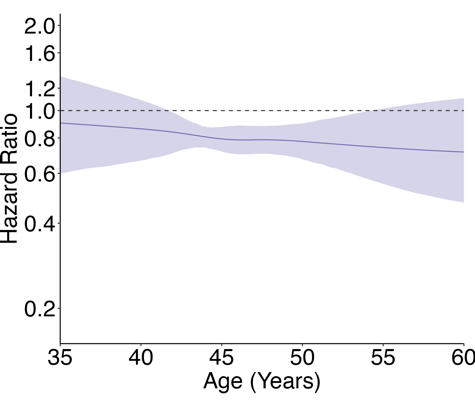 | 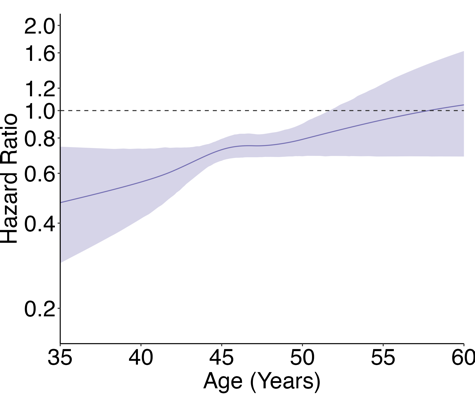 | 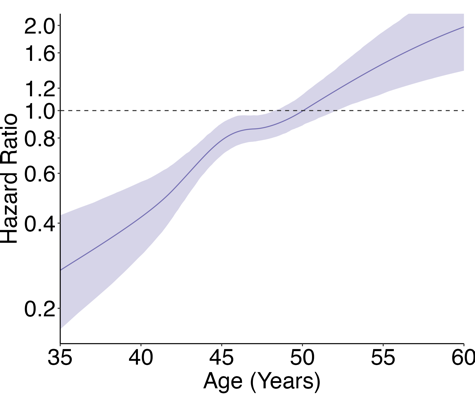 | 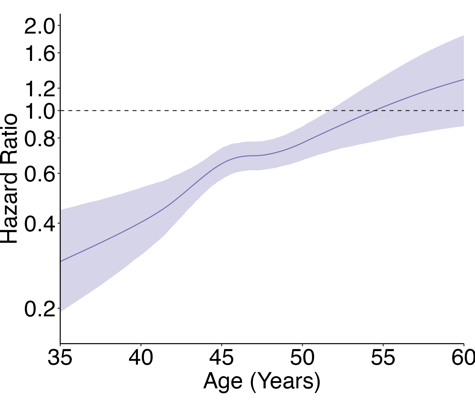 |

1. Restricted to parity of 2 (N = 9,695)

2. Censored at initiation of premenopausal hormone therapy (N = 19,689)

3. Additionally adjusted for body mass index and chronic medical conditions (diabetes, cardiovascular disease, autoimmune disease) (N = 19,431)

4. Restricted to menopause >40 years (N = 18,469)

5. Weighted by inverse probability of censoring weights (N = 19,417)

Models controlled for birth year, education, parity (not applicable for sensitivity analysis 1), duration of hormonal contraceptive use, and smoking.

Y-axes of all figures were restricted to the same range as the HR figures from the main model for better visual comparison.

**Supplemental Figure S4. Sensitivity analyses of the adjusted association between lifetime lactation and timing of indeterminate menopause (premenopausal hysterectomy)**

|  | A. 1-3 months vs. < 1 month | B. 4-6 months vs. < 1 month | C. 7-12 months vs. < 1 month | D. ≥ 13 months vs. < 1 month |
| --- | --- | --- | --- | --- |
| 1. | 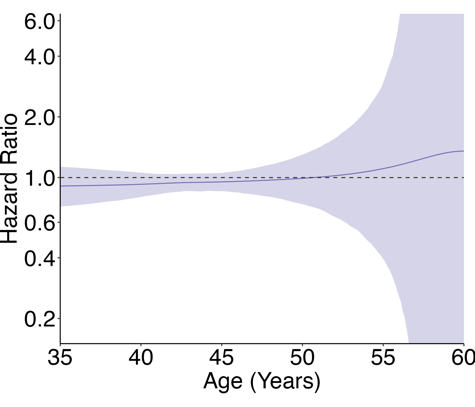 | 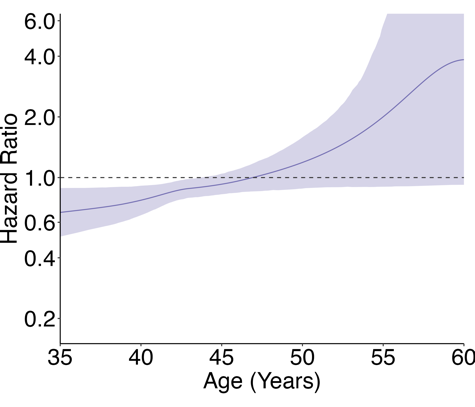 | 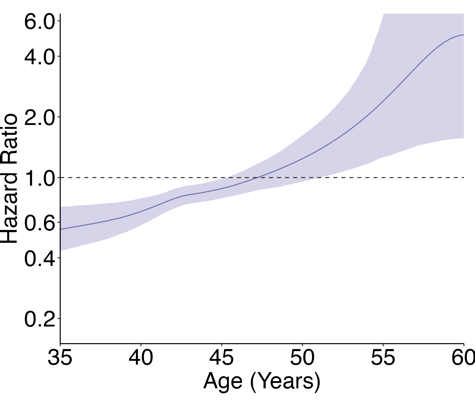 | 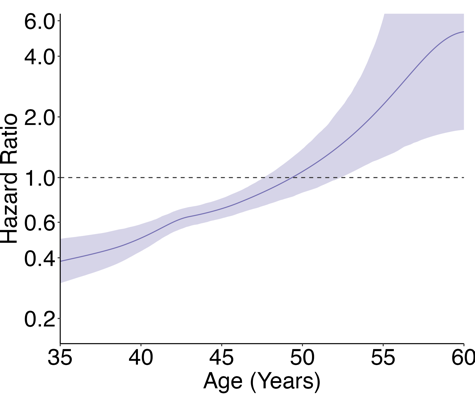 |
| 2. | 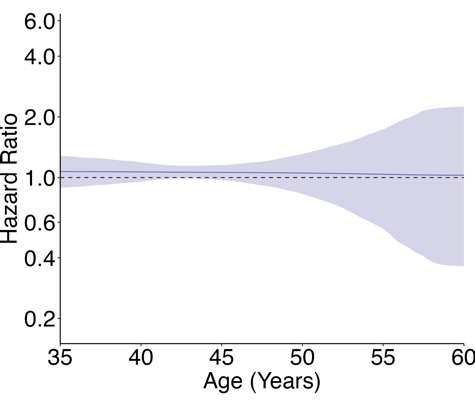 | 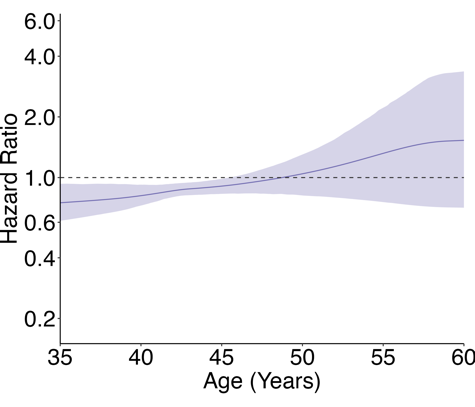 | 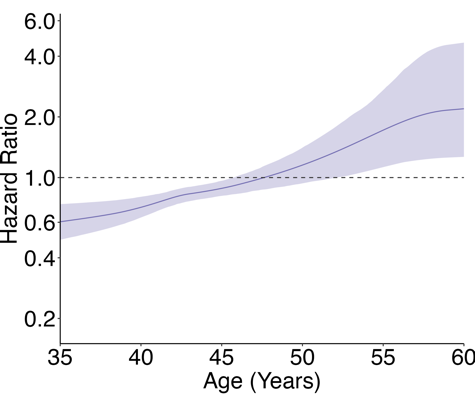 | 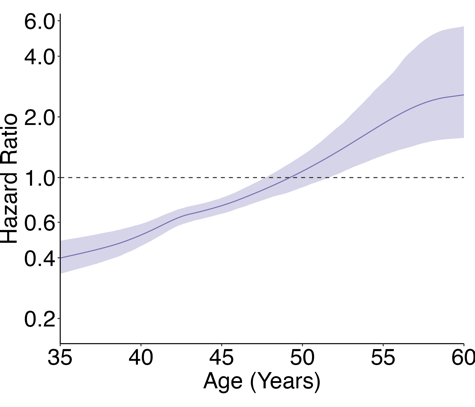 |
| 3. | 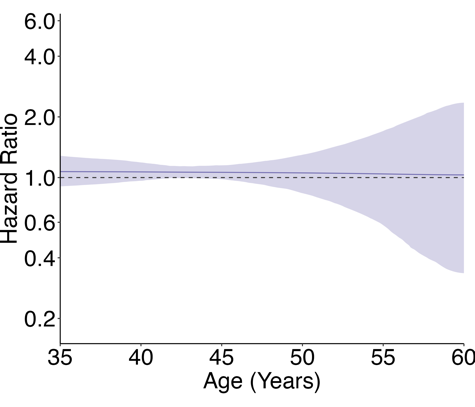 | 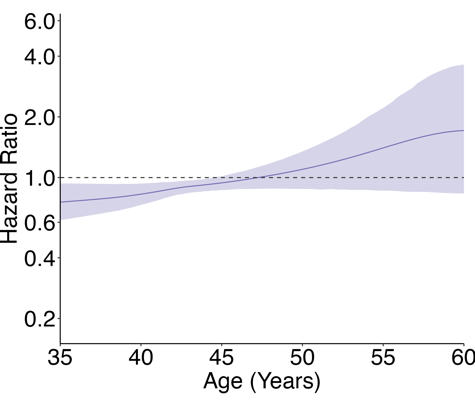 | 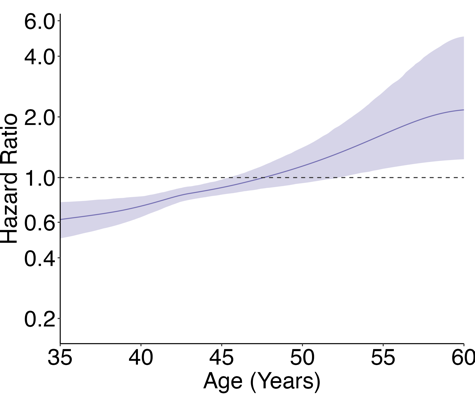 | 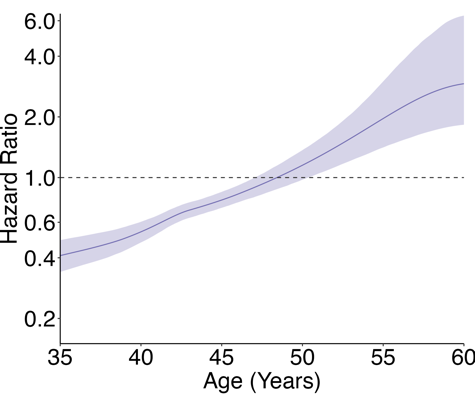 |
| 4. | 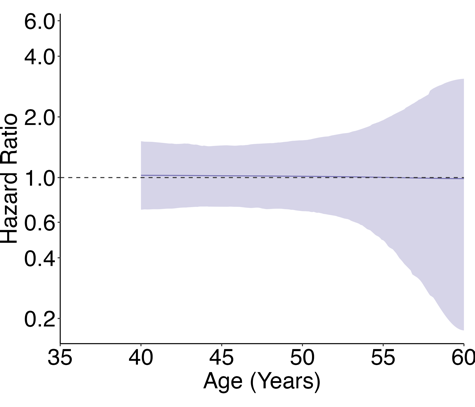 | 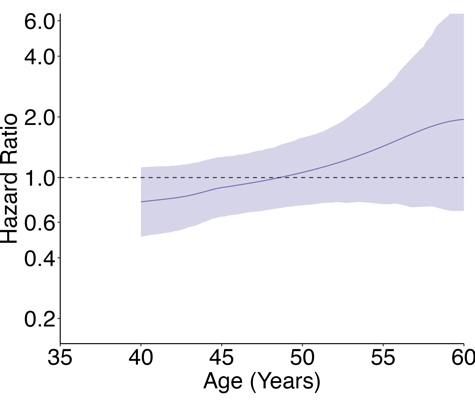 | 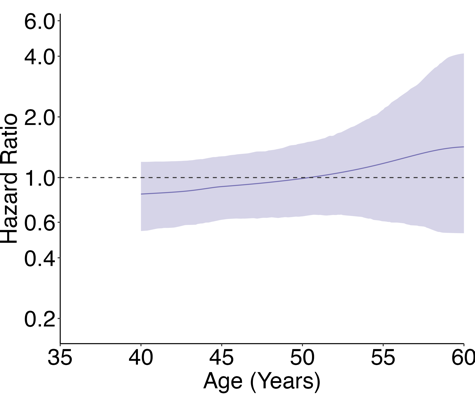 | 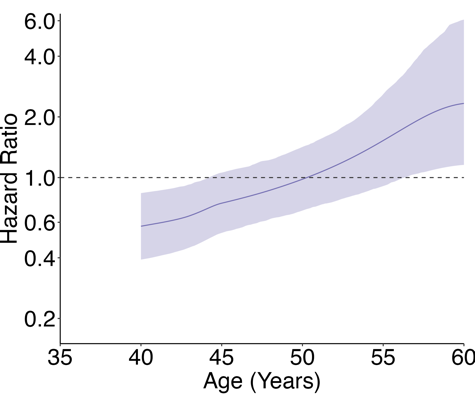 |
| 5. | 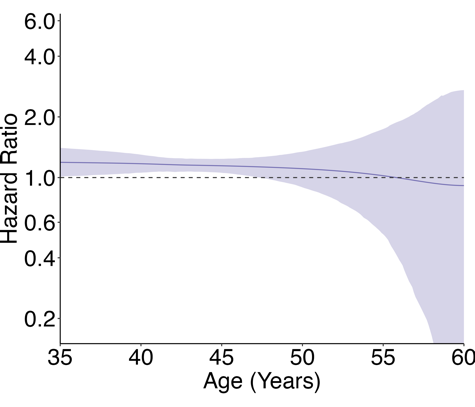 | 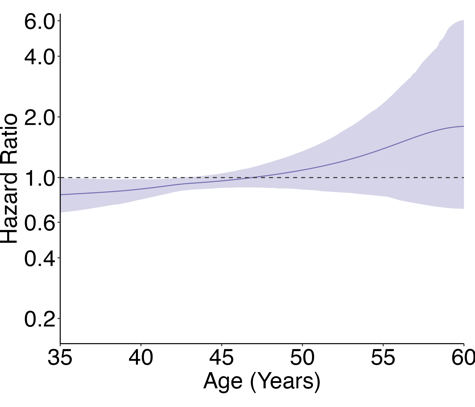 | 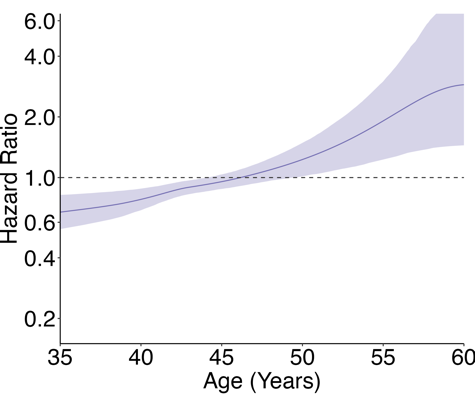 | 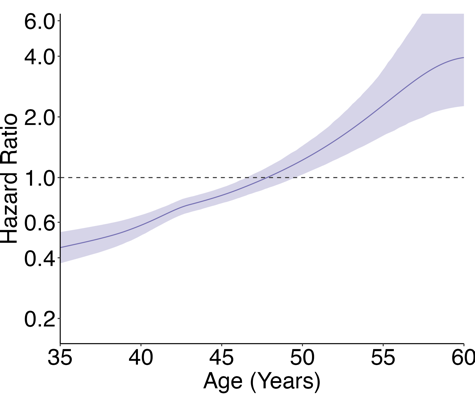 |

1. Restricted to parity of 2 (N = 9,695)

2. Censored at initiation of premenopausal hormone therapy (N = 19,689)

3. Additionally adjusted for body mass index and chronic medical conditions (diabetes, cardiovascular disease, autoimmune disease) (N = 19,431)

4. Restricted to menopause >40 years (N = 18,469)

5. Weighted by inverse probability of censoring weights (N = 19,417)

Models controlled for birth year, education, parity (not applicable for sensitivity analysis 1), duration of hormonal contraceptive use, and smoking.

Y-axes of all figures were restricted to the same range as the HR figures from the main model for better visual comparison.
